# Vascular access for hemodialysis and catheter-related bloodstream infections: a survey on preventive measures and treatment strategies by the EPDWG and ESPN Dialysis Working Group

**DOI:** 10.1007/s00431-025-06703-7

**Published:** 2026-01-07

**Authors:** Sevcan A. Bakkaloğlu, Emre Leventoğlu, Defne Ezgü, Umut Selda Bayrakçı, Kathrin Buder, Nur Canpolat, Andrea Cappoli, Alejandro Cruz, Eiske Dorresteijn, Osman Dönmez, Hakan Erdoğan, Nilüfer Göknar, Isabella Guzzo, Aysun Karabay Bayazıt, Alexander D. Lalayiannis, Germana Longo, Victor López-Báez, Alvaro Madrid, Kashif Mehmood, Hülya Nalçacıoğlu, Lukasz Obrycki, Gönül Parmaksız, Francesco Peyronel, Nikoleta Printza, Dimitar Roussinov, Rina Rus, Dina E. Sallam, Stella Stabouli, Maria Szczepanska, Yılmaz Tabel, Mehmet Taşdemir, Ana Teixeira, Stéphanie Tellier, Nurdan Yıldız, Ariane Zaloszyc, Claus Peter Schmitt, Rukshana Shroff, Alberto Edefonti

**Affiliations:** 1 Department of Pediatric Nephrology, Faculty of Medicine, Gazi University, Ankara, Türkiye; 2 Department of Pediatric Nephrology, Konya City Hospital, Konya, Türkiye; 3 Faculty of Medicine, Başkent University, Ankara, Türkiye; 4 Department of Pediatric Nephrology, Bilkent City Hospital, Ankara, Türkiye; 5 Department of Pediatric Nephrology, University Children’s Hospital Zurich, Zurich, Switzerland; 6 Department of Pediatric Nephrology, Cerrahpaşa Faculty of Medicine, İstanbul University-Cerrahpaşa, Istanbul, Türkiye; 7 Department of Nephrology, Bambino Gesù Children’s Hospital, IRCCS, Rome, Italy; 8 Department of Pediatric Nephrology, University Hospital Vall d’Hebron, Barcelona, Spain; 9 Department of Pediatric Nephrology, Sophia Children’s Hospital, Erasmus Medical Center, Rotterdam, Netherlands; 10 Department of Pediatric Nephrology, Faculty of Medicine, Uludağ University, Bursa, Türkiye; 11 Department of Pediatric Nephrology, Bursa City Hospital, Bursa, Türkiye; 12 Department of Pediatric Nephrology, Faculty of Medicine, İstanbul Medeniyet University, Istanbul, Türkiye; 13 Department of Pediatric Nephrology, Faculty of Medicine, Çukurova University, Adana, Türkiye; 14 Department of Pediatric Nephrology, Birmingham Women’s and Children’s Hospitals NHS Foundation Trust, Birmingham, UK; 15 Department of Pediatric Nephrology, Dialysis and Transplant Unit, Department of Woman and Child Health, Padua University, Padua, Italy; 16 Department of Nephrology, University Hospital Germans Trias I Pujol, Barcelona, Spain; 17 Department Pediatric Nephrology, Sant Joan de Deu University Hospital, Barcelona, Spain; 18 Department of Pediatrics, Children Kidney Center (IPNA Sister Renal Center), Mayo Hospital, Lahore, Pakistan; 19 Department of Pediatric Nephrology, Faculty of Medicine, Ondokuz Mayıs University, Samsun, Türkiye; 20 Department of Nephrology, Kidney Transplantation and Hypertension, The Children’s Memorial Health Institute, Warsaw, Poland; 21 Department of Pediatric Nephrology, Dr Turgut Noyan Training and Research Center, Başkent University, Adana, Türkiye; 22 Nephrology and Dialysis Unit, Meyer Children’s University Hospital, Florence, Italy; 23 Department of Pediatric Nephrology, 1, Department of Pediatrics , Hippokratio General Hospital, Aristotle University, Thessaloniki, Greece; 24 Department of Pediatrics, SBAL Pediatric Diseases, Nephrology and Hemodialysis Clinic, Medical University of Sofia, Sofia, Bulgaria; 25 Department of Nephrology, Faculty of Medicine, University Children’s Hospital Ljubljana, University of Ljubljana, Ljubljana, Slovenia; 26 Department of Pediatrics and Pediatric Nephrology, Faculty of Medicine, Ain Shams University, Cairo, Egypt; 27 Department of Pediatrics, Faculty of Medical Sciencesin Zabrze, Medical University of Silesia, Katowice, Poland; 28 Department of Pediatric Nephrology, Faculty of Medicine, İnönü University, Malatya, Türkiye; 29 Department of Pediatric Nephrology, Faculty of Medicine, İstinye University, Istanbul, Türkiye; 30 Centro Materno-Infantil Do Norte, Centro Hospitalar Do Porto, Porto, Portugal; 31 Department of Pediatric Nephrology and Rheumatolgy, French Reference Center of Rare Renal Diseases (SORARE), CHU Toulouse, Toulouse, France; 32 Department of Pediatric Nephrology, Faculty of Medicine, Marmara University, Istanbul, Türkiye; 33 Department of Pediatric Nephrology, Centre Hospitalier Universitaire (CHU) de Strasbourg, Strasbourg, France; 34 Department of Pediatric Nephology, Centre for Pediatric and Adolescent Medicine, Medical Faculty Heidelberg, University of Heidelberg, Clinics 1, Heidelberg, Germany; 35 Department of Pediatric Nephrology, Great Ormond Street Hospital for Children, NHS Foundation Trust, London, UK; 36 Pediatric Nephrology, Dialysis and Transplant Unit, Fondazione IRCCS Ca’ Granda Ospedale Maggiore Policlinico, Milan, Italy

**Keywords:** Central venous catheter, Catheter-related bloodstream infection, Lock solutions, Pediatric hemodialysis, Vascular access

## Abstract

The choice of vascular access (VA) plays a key role in the success of hemodialysis (HD). Despite their widespread use, central venous catheters (CVCs) are associated with higher rates of dysfunction, thrombosis, and catheter-related bloodstream infections (CRBSI). We investigated current practices in pediatric HD across European pediatric nephrology centers, focusing on VA choices, infection control measures, and CRBSI management. An online questionnaire was e-mailed to 119 members of the European Society for Pediatric Nephrology (ESPN) Dialysis Working Group and European Pediatric Dialysis Working Group (EPDWG). Descriptive statistics were used to summarize practices across centers, comparative analyses between centers in countries with Human Development Index (HDI) > 0.90 and < 0.90. Thirty-one centers across Europe participated in the survey. CVCs were the primary VA in 73.1% of the centers. Twenty (66.7%) centers reported malfunction as the most common CVC complication, followed by catheter thrombosis (19.4%) and CRBSI (12.9%). The diagnostic approach for CRBSI varied widely, with 35.4% of centers relying on a single positive catheter culture, while 57.9% did not collect a second culture from the peripheral vein or HD circuit. The most common empirical treatment was glycopeptides combined with third-generation cephalosporins. Nearly all centers used intravenous antibiotics for less than 3 weeks, and over half modified lock solutions with antibiotics following CRBSI diagnosis. Catheter removal practices were inconsistent, even in cases of severe infection. Centers reported a total of 548 HD patients. Exit-site infections and CRBSI were observed in 98 (17.8%) and 155 (28.2%) patients, respectively. CRBSI rates and CRBSI-related catheter replacements were significantly higher in centers from countries with HDI < 0.90 and in centers without a dedicated pediatric HD unit.

*Conclusion*: The suboptimal adherence to current VA recommendations and wide variability in catheter care practices including the prevention, diagnosis, and management of CRBSI highlight the need for standardized pediatric-specific protocols to enhance catheter longevity and improve patient outcomes.

**Table:** 

**What is Known:** • Central venous catheters are widely used in pediatric hemodialysis but carry a high risk of complications, especially catheter-related bloodstream infections (CRBSI).
**What is New:** • This multinational survey reveals significant variability in vascular access selection, CRBSI prevention, diagnosis, and treatment across European pediatric hemodialysis centers, with clear disparities by national HDI levels. • The findings highlight the need for standardization of vascular access care and CRBSI management and evidence-based pediatric-specific guidelines.

**Supplementary Information:**

The online version contains supplementary material available at 10.1007/s00431-025-06703-7.

## Introduction

Hemodialysis (HD) is a life-sustaining therapy for children with end-stage kidney disease [1]. The management of pediatric HD requires specialized care due to the unique anatomical and physiological characteristics of children, especially in terms of vascular access (VA) and related complications [2]. Although arteriovenous fistulas (AVFs) are associated with superior outcomes compared to central venous catheters (CVCs) and are the recommended VA in children [3–5], recent studies still consistently demonstrate a marked predominance of CVC use over AVFs, despite these guidelines [5–9].

CVCs have a significantly higher rate of dysfunction than AVF, due to malfunctions, thrombosis, and catheter-related bloodstream infections (CRBSI) [3, 5, 7, 10, 11]. An analysis of prospectively collected data in the IPHN identified infectious complications solely associated with CVCs (1.3/1000 catheter days), and 47% of cases required VA replacement [7]. Microbial colonization and biofilm formation on the CVC typically occur through the exit site, healthcare providers’ hands, and catheter hubs [12]. Risk factors for the development of CRBSI include catheter-related causes, such as catheter type, insertion site, and duration of catheter use, and patient-related causes, such as young age, immunodeficiency status, previous bacteremia or CRBSI, and nasal carriage of *Staphylococcus aureus* [13, 14]. Infection prevention and management strategies, including the use of antibiotics, catheter lock solutions, and various techniques for maintaining catheter patency and reducing contamination, are critical for improving patient outcomes [15]. The Standardizing Care to Improve Outcomes in Pediatric End Stage Renal Disease (SCOPE) collaborative in the USA aimed to reduce CRBSIs in pediatric HD patients by improving adherence to standardized catheter care bundles. Over a 48-month period, overall bundle compliance significantly increased, and the adjusted CRBSI rate declined from 3.3 to 0.8 infections per 100 patient-months [16]. These findings highlight the effectiveness of quality improvement strategies in enhancing catheter care and reducing infection rates in this population.

This survey aimed to assess current practices in pediatric nephrology centers that are part of the European Society for Pediatric Nephrology (ESPN) Dialysis Working Group and the European Pediatric Dialysis Working Group (EPDWG), with a specific focus on VA strategies, infection control measures, and management of CRBSI in children to identify potential targets for quality improvement.

## Material and methods

Our online survey consisted of seven sections, and 51 questions were prepared via “Survey Monkey.” The first section included six questions about institutional and professional demographics, and the second included twelve questions about VA types used within the centers. The third section assessed VA-related complications with two questions, the fourth asked twelve questions about measures to prevent CRBSI. The fifth section involved two questions on the diagnosis of CRBSI, the sixth section contained ten questions on systemic treatment of CRBSI, and the seventh section included seven questions on catheter lock solutions. The survey questions are shown in Supplemental Table 1.

**Table 1:** Countries according to the human development Index and the number of centers surveyed from these countries

Countries	The number of centers participated in the survey	HDI[17]
Switzerland	1	0.967
Germany	1	0.950
Netherlands	1	0.946
United Kingdom	2	0.940
Slovenia	1	0.926
Spain	4	0.911
France	2	0.910
Italy	3	0.906
Greece	1	0.893
Poland	1	0.881
Portugal	1	0.874
Turkey	12	0.855
Bulgaria	1	0.799

HDI, Human Development Index

In addition to questions on center characteristics and practices, the survey included a section on patient outcomes. Centers were asked to report the total number of chronic HD patients, the number of patients who developed CRBSI or exit-site infections over the past 3 years, the number of catheter replacements performed, and the indication for each replacement (CRBSI vs. exit-site infection). These data allowed descriptive and comparative analyses of outcomes in relation to center-specific practices.

The online survey was sent via email to pediatric nephrologists who are members of the ESPN Dialysis Working Group and the EPDWG, which has a total of 119 members. A single response, ideally from the HD lead, in each center was requested.

Human Development Index (HDI) is a composite indicator developed by the United Nations Development Program to assess and compare countries’ levels of human development. It combines three key dimensions: health (measured by life expectancy at birth), education, and standard of living (represented by gross national income per capita) [17]. In this study, countries were categorized into groups based on their HDI levels: HDI > 0.90 and HDI < 0.90 (Table 1). This classification was used to evaluate potential associations between human development levels and VA type and preventive measures and treatment strategies for CRBSIs across different regions in Europe.

### Statistical analysis

Descriptive statistics were reported as counts and percentages for categorical variables. Continuous variables were expressed as means with standard deviations if normally distributed, and as medians with interquartile ranges (IQRs) if non-normally distributed. Categorical variables were compared using the chi-square test. Differences between two groups were assessed with Student’s *t*-test for normally distributed data or the Mann–Whitney *U* test for non-normally distributed data. Statistical analyses were performed using IBM SPSS Statistics, Version 25.0, and a *p*-value < 0.05 was considered statistically significant.

## Results

### General study population

A total of 31 centers participated in the survey. Twenty-three (74.1%) of them have a pediatric HD unit. CVC was the most common (73.1%) VA, followed by AVF and AVG (25.8% and 1.1%, respectively). Eighteen (58%) of the centers used only cuffed/permanent, double-lumen CVCs. Almost all centers (*n* = 29, 93.5%) used the right internal jugular vein (IJV) access. Twenty-one (67.7%) participants reported malfunction as the most common CVC complication, followed by thrombosis and CRBSI (19.4% and 12.9%, respectively) (Table 2).

**Table 2:** The results of the survey on vascular access

	General group (*n* = 31)			
	*n*	%	Mean (SD)	Min–max
**Type of VA**				
Percentage of maintenance HD with CVC			73.1 (23.8)	20–100
Percentage of maintenance HD with AVF			25.8 (22.7)	0–80
Percentage of maintenance HD with AVG			1.1 (4.4)	0–20
**Type of CVC in maintenance HD**				
Only cuffed permanent double-lumen CVC	18	58.1		
Mostly cuffed permanent double-lumen CVC	11	35.5		
**Location of permanent CVC in maintenance HD**				
Right internal jugular vein	29	93.5		
Left internal jugular vein	1	3.2		
Subclavian vein	1	3.2		
**Most common VA-related complication**				
CVC malfunction	21	67.7		
CVC thrombosis	6	19.4		
CRBSI	4	12.9		

*VA*, vascular access; *HD*, hemodialysis; *CVC*, central venous catheter; *AVF*, arteriovenous fistula; *CRBSI*, catheter-related blood stream infection

Infection prevention measures by centers are shown in Table 3. Twenty-two centers (71%) administered intravenous antibiotics before the placement of permanent CVCs. Sterile gauze was most frequently used as exit-site dressing (*n* = 13, 41.9%). Chlorhexidine with > 0.5% alcohol solution was used for exit-site antisepsis, hand cleansing, and scrubbing the CVC hubs in approximately half of the centers (*n* = 14 (45.2%), *n* = 17 (54.8%), and *n* = 15 (48.4%), respectively). To prevent CVC contamination, 35.5% of the centers (*n* = 11) used needle-free connectors and only 16.1% (*n* = 5) chlorhexidine-embedded caps. Exit-site ointments were used in 14 centers (45.2%), of which 13 (92.8%) used mupirocin.

**Table 3:** The survey results about infection prevention measures

	General group (*n* = 31)	
	*n*	%
**Use of intravenous antibiotics before the insertion of long-term CVCs to prevent CRBSI**	22	71
**Dressings for CVC exit site**		
Transparent semipermeable dressing	11	35.5
Sterile gauze dressing	13	41.9
Chlorhexidine impregnated patch with transparent semipermeable dressing	7	22.6
**Frequency of exit-site cleaning**		
Once weekly	16	51.6
Each HD session	15	48.4
**Antisepsis for exit site**		
> 0.5% chlorhexidine with alcohol solution	14	45.2
70% alcohol solution	2	6.5
10% povidone–iodine solution	10	32.3
**Antisepsis before handling**		
> 0.5% chlorhexidine with alcohol solution	17	54.8
70% alcohol solution	8	25.8
10% povidone–iodine solution	4	12.9
**Antisepsis for CVC hubs**		
> 0.5% chlorhexidine with alcohol solution	15	48.4
70% alcohol solution	4	12.9
10% povidone–iodine solution	8	25.8
Chlorhexidine gluconate-based surgical scrubs	4	12.9
**Use of chlorhexidine embedded cap device for CVC**	5	16.1
**Use of needle-free HD connector for preventing CVC contamination**	11	35.5
**Use of exit-site ointments**	14	45.2
Mupirocin	13	92.8

*CVC*, central venous catheter; *CRBSI*, catheter-related blood stream infection; *HD*, hemodialysis

The results regarding the diagnosis and management of CRBSI are shown in Table 4. The most common causes of CRBSI were reported to be immunodeficiency status (*n* = 14, 45.1%), history of previous CRBSI (*n* = 12, 38.7%), and malnutrition (*n* = 12, 38.7%). For the diagnosis of CRBSI, some centers (*n* = 11, 35.4%) considered one positive culture from the CVC lumen sufficient. Eighteen (58%) centers did not take a second culture from the peripheral vein or HD circuit. Additionally, 12 centers (38.7%) reported that they do not routinely consider the differential time to positivity in the evaluation of suspected CRBSI.

**Table 4:** The results of the survey related to the diagnosis and management of CRBSI

	General group (*n* = 31)	
	*n*	**%**
**Risk factors for CRBSIs**		
Young age	11	35.4
Body weight	5	16.1
Catheter location other than right Internal jugular vein	7	22.5
Immunodeficient state	14	45.1
Malnutrition	12	38.7
Prior CRBSI of the current catheter	12	38.7
Prior systemic antibiotic use (not related to CRBSI)	1	3.2
Nasal carriage of Staphylococcus aureus	7	22.5
**Number of cultures required for CRBSI diagnosis**		
One positive culture form CVC	11	35.4
Two positive cultures from the two lumens of CVC	7	22.5
Two positive cultures; one from CVC and one from peripheral vein	12	38.7
Two positive cultures; one from CVC and one from HD circuit	1	3.2
**Use of differential time of culture positivity to detect CRBSI**	18	58
**Empiric antibiotic selection**		
Glycopeptide	9	29
Glycopeptide and third-generation cephalosporin	12	38.7
Others	10	32.2
**Measurement of antibiotic levels**		
Vancomycin level	21	67.7
Aminoglycoside level	20	64.5
**Duration of antibiotic treatment for CRBSI**		
7–10 days	8	25.8
2 weeks	14	45.1
> 2–3 weeks	6	19.3
3 weeks	3	9.6
**Use of antibiotic lock solutions (and anticoagulant) in addition to systemic antibiotics**	18	58
**Use of anti-fungal prophylaxis during treatment of bacterial CRBSI**	6	19.3
Nystatin	2	33.3
Fluconazole	4	66.7
**Indications of catheter removal**		
Severe sepsis/septic shock	19	61.2
Metastatic infection (endocarditis, septic arthritis)	20	64.5
Infection with *S. aureus*	6	19.3
Infection with *P. aeruginosa*	16	51.6
Infection with fungi	29	93.5
Infection with multi-resistant organism	14	45.1
Persistently positive blood cultures	27	87.1
Tunnel infection	19	61.2

CRBSI, catheter-related blood stream infection; CVC, central venous catheter; HD, hemodialysis

The most common empirical treatment combination for CRBSI was glycopeptides with third-generation cephalosporins (*n* = 12, 38.7%). If methicillin-sensitive *Staphylococcus aureus* (MSSA) grew in culture, most participants (*n* = 21, 67.7%) reported to switch from empiric glycopeptide to cefazolin. In CRBSI, nearly all centers (*n* = 28, 90.3%) used an intravenous antibiotic regimen of less than 3 weeks, most commonly for 2 weeks (45.1%) or 7–10 days (25.8%), while only a small proportion extended treatment to 3 weeks (9.6%). To optimize CRBSI management, 18 centers (58%) reported modifying their standard lock solutions by adding antibiotics following CRBSI diagnosis, in addition to administering systemic antibiotic therapy. In cases of CRBSI caused by *Staphylococcus aureus* and *Pseudomonas aeruginosa*, catheter removal was not routinely performed in 80.6% and 48.3% of centers, respectively. Also, 54.8%, 38.7%, 38.7%, and 35.4% of centers reported not to remove the catheter in cases of multi-resistant microorganisms, tunnel infections, sepsis/septic shock, and metastatic infections, respectively.

All centers routinely used antibiotic-free catheter lock solutions, except during episodes of infections. Twenty-two centers (71%) used a single agent as lock solution, with heparin being the most frequent (*n* = 17, 54.8%). The 2 + 1 protocol—where one lock solution combination is used after two dialysis sessions and a different combination after the third session each week—was used in 4 (12.9%) centers. Among these, 3 centers (75%) followed a regimen consisting of taurolidine/citrate/urokinase 25,000 U/mL once weekly and taurolidine/citrate/heparin 500 U/mL twice weekly (Table 5).

**Table 5:** The results of the survey about catheter lock solutions

	General group (*n* = 31)	
	*n*	%
**Routine use of catheter lock solutions**		
**Single agent**	21	67.7
Heparin 1000	8	38.1
Heparin 2000	2	9.5
Heparin 5000	7	33.3
rt-PA	2	9.5
Urokinase	1	4.7
Citrate	1	4.7
**Double combination**	6	19.4
Tauridin + citrate	2	33.3
Heparin + antibiotic	4	66.7
**Triple combination**	3	9.7
Tauridin + citrate + heparin 500	3	100
**Use of 2 + 1 protocols for lock solutions**	4	12.9
Taurolidine/citrate/heparin 500 U twice a week and taurolidine/citrate/urokinase 25,000 U once a week	3	75
Heparin 5000 U twice a week and rt-PA once a week	1	25

rt-PA, recombinant tissue plasminogen activator

### Clinical practice according to the country’s HDI

Fifteen centers (48.3%) had an HDI > 0.90, while sixteen centers (51.6%) had an HDI < 0.90. Comparisons based on countries’ HDI revealed important disparities. While all centers in countries with HDI > 0.9 had a pediatric-specific HD unit, this was the case in only 50% of centers in countries with HDI < 0.90 (*p* = 0.002). In 8 out of 16 centers, children are followed by pediatric nephrologists but undergo dialysis in a combined unit shared with adults. Although not statistically significant, the CVC rate was lower and the AVF rate was higher in centers in high-HDI countries [68.2% vs. 77.5% (*p* = 0.286) and 30.7% vs. 21.1% (*p* = 0.250)]. In countries with HDI < 0.90, 62.5% of centers reported use of non-cuffed tunneled catheters for chronic HD, while centers of countries with an HDI > 0.90, 80% use only cuffed tunneled catheters (*p* = 0.044).

Exit-site cleansing was performed every dialysis session in 75% of centers in countries with low HDI, whereas it was performed once weekly in 80% of centers in high-HDI countries (*p* = 0.003). For hand antisepsis, 10% povidone–iodine was used in 25% of centers of low-HDI countries, but in none of the centers from higher-HDI countries (*p* = 0.028). In low-HDI countries, sterile gauze was employed in 62.5% of centers, whereas transparent semipermeable dressing was preferred in 60% of centers in high-HDI countries (*p* = 0.015). The use of needle-free HD connectors was significantly higher in high-HDI countries (60%) than in low-HDI countries (12.5%) (*p* = 0.006).

In the diagnosis of CRBSI, two cultures were taken in 68.8% of low-HDI centers, one from the CVC lumen and the other from the peripheral vein/HD circuit, while this rate was 13.3% in centers with high HDI (*p* = 0.007). There was no difference in the choice of empirical antibiotic therapy between the two HDI groups. Centers in low-HDI countries less frequently monitored plasma vancomycin and amikacin concentrations [43.3% vs 93.3% (*p* = 0.003) and 43.8% vs 86.7% (*p* = 0.013), respectively].

Almost 50% of the centers (46.7%) in high-HDI countries used double or triple agents, while most centers (87.5%) in countries with lower HDI used single agents (*p* = 0.029) as routine catheter lock solution. Heparin alone was used in 87.5% of centers in countries with HDI < 0.90 compared to 20% with HDI > 0.90 (*p* = 0.001). Among centers using dual or triple agents, a heparin–antibiotic combination was employed in 25% of centers in low-HDI countries, while no center in high-HDI countries routinely included antibiotics in lock solutions (*p* = 0.006). Furthermore, the 2 + 1 protocol was not used in any center with HDI < 0.90 (*p* = 0.040).

### Comparison of patient outcomes based on center characteristics

The total number of HD patients at the centers was 548. A total of 351 (64%) patients underwent HD with a permanent-cuffed catheter. There were 98 (17.8%) patients with reported exit-site infections and 155 (28.2%) patients with CRBSI. A total of 213 (38.8%) patients underwent CVC replacement. Of these replacements, 122 (57.2%) were due to CRBSI and 19 (8.9%) were due to exit-site infection.

According to analyses based on the HDI, the median rate of CRBSI in centers in countries with an HDI < 0.90 was significantly higher compared to centers in countries with an HDI > 0.90 (40.58% vs. 10.35%, *p* = 0.001). As a result, the frequency of CVC change due to CRBSI was also higher in these centers (20.34% vs. 6.90%, *p* = 0.015). In analyses based on whether the center was a pediatric HD center, the incidence of CRBSI was higher in centers without a pediatric HD unit (50.0% vs. 18.33%, *p* < 0.001), and the rate of catheter replacement due to CRBSI was also higher in these centers (36.66% vs. 10.0%, *p* = 0.002). Furthermore, the overall CVC replacement rate was higher in centers without a pediatric HD unit than in centers with a pediatric HD unit (65.0% vs. 16.66%, *p* = 0.016). In analyses related to the use of needle-free connectors, the incidence of CRBSI was lower in centers using these connectors compared to centers not using them (8.33% vs. 30.45%, *p* = 0.046).

In comparisons between other center characteristics and practices, no statistically significant differences were observed in terms of CRBSI and CVC replacement rates.

## Discussion

Vascular access choices and CRBSIs continue to present a significant challenge in pediatric HD, despite significant progress in infection control and VA management strategies. This study demonstrated a predominant reliance on CVCs and a notable heterogeneity in infection prevention protocols and CRBSI management across European pediatric HD centers. Differences in both routine practices and management of CRBSI underscored the importance of standardized protocols based on evidence-based data to optimize care quality and patient outcomes.

The CVCs were shown as a widely used primary VA in pediatric HD due to the ease of placement and suitability for small children [3, 4, 6, 7]. The incidence of CVC was high in all centers in our study, and the overall rate was 73.1%, in line with the previous studies. In pediatric HD patients in Catalonia, 56.5% started dialysis with AVF between 1997 and 2001, in contrast to no patients with AVF between 2012 and 2018 [18]. Despite a well-known a higher incidence of CVC infection compared to AVF [5, 7, 19], CVC placement rate also increased from 8.7 to 72.2% during this period [18].

Catheter-related infections necessitate access revisions and changes in dialysis modalities, longer hospitalizations, higher morbidity and mortality, a reduction in quality of life, and higher healthcare costs [5, 9, 12, 16]. Although tunneled catheters protect against infections by preventing peri-catheter bacterial entry into the bloodstream, antibiotic prophylaxis before catheter insertion is controversial [20]. In a Cochrane review, six trials compared the use of antibiotics (vancomycin, teicoplanin, ceftazidime, or cefazolin) versus no antibiotics before inserting a long-term CVC. The analysis found no significant reduction in gram-positive CVC-related infections (pooled risk ratio 0.67, 95% CI 0.32 to 1.43), with infection rates of 7.3% vs 10.4%. They concluded that antibiotic administration before CVC implantation has no role in preventing CRBSI [21]. However, it should be noted that most of the centers in our study performed prophylactic antibiotics before implantation of CVCs.

Training both patients and HD staff about the risks associated with long-term catheter use and the best practices for catheter care is crucial in reducing CRBSI. Adhering to established protocols for maximal barrier precautions and strict compliance with universal aseptic techniques when handling catheters is essential [12, 22]. Pediatric studies have shown that chlorhexidine with alcohol is more effective than povidone–iodine in preventing exit-site infections [23]. Transparent semipermeable dressings or chlorhexidine-impregnated patches can be used, with the current practice being weekly changes of the patches [24]. The use of antimicrobial ointments during dressing changes is recommended. Triple antibiotic ointments such as bacitracin, gramicidin, and polymyxin B ointments are superior to mupirocin due to increasing resistance to mupirocin [11, 12]. In our study, the exit-site cleansing was done weekly in slightly more than half of the centers. Nearly half of the centers used a chlorhexidine-alcohol solution for hand antisepsis, followed by alcohol and povidone–iodine. Also, most centers used mupirocin ointment for exit-site care. Despite existing recommendations, the use of povidone–iodine and mupirocin ointment is worth noting and once again demonstrates that an update and standardization are still needed in our traditional practices.

To prevent catheter hub contamination, the use of chlorhexidine gluconate–based surgical scrubs instead of alcohol-based ones is recommended. Recently introduced hub devices containing chlorhexidine may help reduce CRBSIs in high-risk dialysis patients who are prone to recurrent CRBSI, particularly in centers with uncontrolled infection rates [25]. The KDOQI suggests using antimicrobial barrier caps to decrease CRBSI in high-risk patients or facilities, with the choice of needle-free HD connector [26]. Chlorhexidine-embedded rod devices are innovative tools for CRBSI prevention; they gradually release chlorhexidine into the catheter lock solution and effectively eliminate microbes near the hub [27]. In our study, the preferred solution for CVC hub cleansing was also chlorhexidine with alcohol. In addition, many centers used needle-free connectors to prevent CVC contamination, while a few employed chlorhexidine-embedded caps.

An important finding from our study is the variability in the strategies for diagnosing CRBSI. Some centers required two positive blood cultures, one from the CVC and one from a peripheral venous system, while others relied on a single positive culture from the CVC lumen. Additionally, in some centers, even if two different cultures are taken, no attention is paid to the differential time between the growths in the cultures. Key diagnostic criteria for CRBSI include differential culture positivity, isolation of the same microorganism from the catheter and peripheral or circuit samples, and a catheter culture growing ≥ 2 h earlier than the others, suggesting a catheter-related source [25, 27–29]. This variability in our study highlights the need for standardized CRBSI diagnostics, as inconsistent practices may delay treatment and worsen outcomes. Notably, none of the centers utilized HD circuit cultures, despite the importance of preserving peripheral veins in chronic HD patients and the potential of circuit sampling as a reasonable alternative for diagnosing CRBSI.

Antibiotic treatment of CRBSI is often initiated empirically based on the severity of the patient’s condition, risk factors, and the likely pathogens. Glycopeptides are commonly recommended in areas with high rates of methicillin-resistant *Staphylococcus aureus* (MRSA)*.* Empirical coverage for enteric gram-negative bacilli, such as with a third-generation cephalosporin, should also be included to minimize the risks. Once the pathogen is identified, rather targeted/specific antimicrobial therapy according to resistance pattern should be started [30]. Although the recommended duration of treatment is 3 weeks [31], recent evidence suggests a 2-week course of effective antibiotic therapy with the exceptions of *Pseudomonas aeruginosa*, *Acinetobacter baumanii*, and *Stenotrophomonas maltophilia*, which may also be trialed for CRBSI management [32, 33]. In CRBSIs, the chance of success with systemic antibiotic treatment alone is 30–45% and the risk of relapse is high. Therefore, adjuvant antibiotic lock with the same antibiotic may be used to eradicate catheter biofilm and permit effective clearance of the bacteria [34]. Catheter removal should also be considered an additional step alongside systemic antibiotic therapy for managing severe complications, such as sepsis, suppurative thrombophlebitis, persistent bloodstream infections, or ongoing clinical signs of infection despite 48–72 h of effective antimicrobial treatment. This also applies to infections caused by *Staphylococcus aureus*, *Pseudomonas aeruginosa*, multi-resistant organisms, fungi, and tunnel infections accompanied by fever [35]. In an Italian survey, when CRBSI was caused by *Staphylococcus aureus* or *Pseudomonas aeruginosa*, removal or replacement of the CVC was performed only when effective antibiotic treatment failed [36]. In our survey, the treatment strategies for CRBSI exhibited some considerable variations. The use of empirical antibiotics including glycopeptides combined with third-generation cephalosporins was common; however, the duration of treatment and management of catheter removal continue to be a major challenge. Treatment duration was shorter in many centers. When fungal infection was detected, almost all centers removed the CVC. In the case of persistent growth on cultures, most respondents also reported catheter removal, although in cases of sepsis/septic shock, metastatic infections, and tunnel infections, the proportion was no more than three quarters. Since there is no existing evidence based on randomized controlled trials, management of CRBSI is not uniform and commenting initial antibiotic strategies are variable.

The role of catheter lock solutions in preventing CRBSI is another critical area of practice. A meta-analysis showed that antimicrobial lock solutions reduced the rate of CRBSI by 69% and the rate of exit-site infections by 32% [37]. In a study, the CRBSI rate in the ethanol and heparin group was 7.69%, while it was 21.6% in the heparin-only group [38]. Also, taurolidine and heparin lock solutions have been shown to reduce the risk of CRBSI by 71% [39]. However, heparin-only solutions may counteract the bactericidal effects of certain antibiotics and promote biofilm formation [40, 41]. Fortunately, low concentrations of citrate have antimicrobial or biofilm-clearing properties. Indeed, 4% citrate has shown superior efficacy compared to heparin [42] and recommended by the American Society for Diagnostic and Interventional Nephrology [43]. The routine use of antibiotic lock solutions outside of CRBSI episodes is not recommended, as it may contribute to antibiotic resistance and predispose patients to more severe infections [44]. Despite this recommendation, some centers in our study reported the routine use of lock solutions containing antibiotic-containing agents. Given the development of resistance, it may be more appropriate to restrict the use of antibiotic lock solutions to CRBSI episodes. Therefore, the lack of uniformity in protocols highlights the need for further research and the development of standardized guidelines to optimize clinical practice.

Despite advances in catheter care, there is still significant variability in practices due to clinical experience, institutional resources, and local guidelines [45]. Therefore, our findings revealed marked differences in VA practices, infection prevention protocols, and CRBSI management strategies between pediatric HD centers in countries with different HDI levels. Centers in high-HDI countries were more likely to have pediatric-specific HD units, use cuffed double-lumen catheters, adopt evidence-based antisepsis methods, and routinely apply advanced infection prevention measures such as needle-free connectors and transparent dressings. For example, CVC prevalence was lower in countries with HDI < 0.90 than in countries > 0.90 because of the higher AVF placement rates in the latter. These differences may reflect variations in healthcare infrastructure, patient population characteristics, and medical training. Also, we speculate that it may be partly related to specialized and dedicated vascular surgeons with microsurgical skills, which are more common in countries with HDI > 0.90 [46–48]. Conversely, practices in low-HDI countries often reflected resource limitations, including the frequent use of non-cuffed catheters, povidone–iodine, and sterile gauze. In addition, significant disparities were observed in the diagnosis and management of CRBSI, with centers in high-HDI countries more consistently tailoring antibiotic therapy to the narrowest effective spectrum and monitoring plasma drug levels. Observed marked differences in pediatric HD care across Europe underscore the need for systematic tracking of CRBSI episodes, root cause analyses, and well-designed controlled trials to develop evidence-based protocols aimed at improving adherence to prevention and treatment strategies, as well as training programs to support centers.

Analyses of center-specific patient outcomes indicate that CRBSI and CVC replacement rates are significantly associated with center characteristics such as HDI level and the presence of a pediatric HD unit. Specifically, CRBSI rates and catheter replacements due to CRBSI are markedly higher in countries with an HDI < 0.90 and in centers without a pediatric HD unit. In contrast, CRBSI rates were found to be lower in centers using needle-free connectors. These findings once again emphasize that differences in practices between centers can have significant effects on patient outcomes and underscore the importance of standardized protocols.

This study has several limitations. First, it relies on self-reported data, which may be subject to reporting bias or inaccuracies. Second, the relatively low participation rate could have introduced a selection bias, favoring more advanced and better-resourced centers. As in other survey-based studies, practices in less-resourced or less-engaged units likely remain underrepresented, leaving important aspects of routine care in these settings unknown. Additionally, a disproportionately high number of participating centers from Türkiye (12 out of 31 centers) might influence the results and limit the generalizability of the findings to a broader international context. In addition, the total number of HD patients at each center, the frequency of CRBSI and exit-site infections, and catheter replacement rates were collected and analyzed. However, since individual patient characteristics (age, weight, proximity to transplantation, etc.) could not be evaluated, patient-level factors that could influence CVC or AVF usage preferences between some centers could not be fully reflected. The survey’s cross-sectional design hinders the ability to draw causal conclusions regarding the effectiveness of different preventive and treatment strategies. Nonetheless, the insights gained provide a valuable snapshot of current practices in pediatric HD in Europe and highlight areas for improvement and joint action.

## Conclusion

The effective management of VA and the prevention of CRBSI remain central challenges in pediatric HD. Ensuring catheter longevity and high-quality care requires consistent adherence to infection control protocols, accurate diagnostic approaches, and rational antimicrobial use. The existing variations in clinical practice underscore the importance of developing clear, standardized guidelines with conditional to strong recommendations that can be adapted across diverse settings. Significant improvement in VA and CRBSI practices can only be achieved through coordinated initiatives driven by scientific societies or dedicated working groups.

## Supplementary Information

Below is the link to the electronic supplementary material.

Supplementary file1 (DOCX 18 kb)

## Data Availability

The datasets generated and/or analyzed during the current study are available from the corresponding author upon reasonable request.

## References

[1] Fischbach M, Edefonti A, Schröder C, Watson A, European Pediatric Dialysis Working Group (2005) Hemodialysis in children: general practical guidelines. Pediatr Nephrol 20(8):1054–1066 15947992 10.1007/s00467-005-1876-y PMC1766474

[2] Fadel FI, Abdel Mooty HN, Bazaraa HM, Sabry SM (2008) Central venous catheters as a vascular access modality for pediatric hemodialysis. Int Urol Nephrol 40(2):489–496 17978856 10.1007/s11255-007-9259-x

[3] Ma A, Shroff R, Hothi D et al (2013) A comparison of arteriovenous fistulas and central venous lines for long-term chronic haemodialysis. Pediatr Nephrol 28(2):321–326 23052655 10.1007/s00467-012-2318-2

[4] Shroff R, Calder F, Bakkaloğlu S et al (2019) Vascular access in children requiring maintenance haemodialysis: a consensus document by the European Society for Paediatric Nephrology Dialysis Working Group. Nephrol Dial Transplant 34(10):1746–1765 30859187 10.1093/ndt/gfz011

[5] Bakkaloğlu SA, Özdemir Atikel Y, Schmitt CP et al (2024) Comparative analysis of hospitalizations among patients treated with hemodialysis and peritoneal dialysis in European pediatric nephrology centers: results from a prospective EPDWG/ESPN Dialysis Working Group study. Clin Kidney J 17:1–11 38223336 10.1093/ckj/sfad291 PMC10784969

[6] Boehm M, Bonthuis M, Noordzij M et al (2019) Hemodialysis vascular access and subsequent transplantation: a report from the ESPN/ERA-EDTA registry. Pediatr Nephrol 34(4):713–721 30588548 10.1007/s00467-018-4129-6 PMC6394682

[7] Borzych-Duzalka D, Shroff R, Ariceta G et al (2019) Vascular access choice, complications, and outcomes in children on maintenance hemodialysis: findings from the International Pediatric Hemodialysis Network (IPHN) Registry. Am J Kidney Dis 74(2):193–202 31010601 10.1053/j.ajkd.2019.02.014

[8] Hayes WN, Watson AR, Callaghan N et al (2012) Vascular access: choice and complications in European paediatric haemodialysis units. Pediatr Nephrol 27(6):999–1004 22205507 10.1007/s00467-011-2079-3

[9] Atikel YÖ, Schmitt CP, Lévai E et al (2023) The effects of hospital and dialysis unit characteristics on hospitalizations for access-related complications among children on maintenance dialysis: a European, multicenter, observational, cross-sectional study. Pediatr Nephrol 38(7):2189–2198 36595069 10.1007/s00467-022-05842-5

[10] Chand DH, Swartz S, Tuchman S et al (2017) Dialysis in children and adolescents: the pediatric nephrology perspective. Am J Kidney Dis 69(2):278–286 27940060 10.1053/j.ajkd.2016.09.023

[11] Ruebner RL, De Souza HG, Richardson T et al (2022) Epidemiology and risk factors for hemodialysis access-associated infections in children: a prospective cohort study from the SCOPE collaborative. Am J Kidney Dis 80(2):186–195 34979159 10.1053/j.ajkd.2021.11.008

[12] Bakkaloğlu SA (2023) Central venous catheter-related bloodstream infections in children on maintenance haemodialysis: prevention and treatment. An Nefrol Pediátr 1(6):165–172

[13] Onder AM, Somer MJG (2021) Infectious complications of hemodialysis in children. In: Warady BA, Schaefer F, Alexander SR (eds) Pediatric dialysis, 3rd edn. Springer, Switzerland-Cham, pp 401–436

[14] Onder AM, Chandar J, Coakley S et al (2006) Predictors and outcome of catheter-related bacteremia in children on chronic hemodialysis. Pediatr Nephrol 21(10):1452–1458 16897007 10.1007/s00467-006-0130-6

[15] Weldetensae MK, Weledegebriel MG, Nigusse AT et al (2023) Catheter-related bloodstream infections and associated factors among hemodialysis patients in a tertiary care hospital. Infect Drug Resist 16:3145–3156 37249964 10.2147/IDR.S409400 PMC10216862

[16] Marsenic O, Rodean J, Richardson T et al (2020) Tunneled hemodialysis catheter care practices and bloodstream infection rate in children: results from the SCOPE collaborative. Pediatr Nephrol 35(1):135–143 31654224 10.1007/s00467-019-04384-7

[17] United Nations Development Programme. Human Development Index. Available at: https://hdr.undp.org/data-center/human-development-index#/indicies/HDI (Access date: 04.04.2025)

[18] Roca-Tey R, Ariceta G, Ríos H et al (2024) Changes in vascular access profile for pediatric hemodialysis patients over time: a registry-based study from Catalonia. J Vasc Access 25(6):1989–1995 37817674 10.1177/11297298231202634

[19] Chotikanatis K, Suman N, Bäcker M et al (2015) Pediatric fistula initiative: reducing bloodstream infections in an outpatient pediatric hemodialysis center. J Pediatr Infect Dis Soc 4(4):363–366 10.1093/jpids/piu053 26582876

[20] Sohail MA, Vachharajani TJ, Anvari E (2021) Central venous catheters for hemodialysis—the myth and the evidence. Kidney Int Rep 6(12):2958–2968 34901568 10.1016/j.ekir.2021.09.009 PMC8640568

[21] Van den Bosch C, Van Voensel J, Van de Vetering M (2021) Prophylactic antibiotics for preventing gram-positive infections associated with long-term central venous catheters in adults and children receiving treatment for cancer. Cochrane Database Syst Rev 10(10):CD003295 34617602 10.1002/14651858.CD003295.pub4 PMC8495768

[22] Centers for Disease Control and Prevention. Dialysis Safety Core Interventions. 2016. Available at: https://www.cdc.gov/dialysis/prevention-tools/core-interventions.html (Access date: 16.04.2025)

[23] Onder AM, Chandar J, Billings A et al (2009) Chlorhexidine-based antiseptic solutions effectively reduce catheter-related bacteremia. Pediatr Nephrol 24(9):1741–1747 19296135 10.1007/s00467-009-1154-5

[24] Fischer M, Golestaneh L, Allon M et al (2020) Prevention of bloodstream infections in patients undergoing hemodialysis. Clin J Am Soc Nephrol 15:132–151 31806658 10.2215/CJN.06820619 PMC6946076

[25] Lok CE, Huber TS, Lee T, Shenoy S, Yevzlin AS, Abreo K, Allon M, Asif A, Astor BC, Glickman MH, Graham J (2020) KDOQI clinical practice guideline for vascular access: 2019 update. Am J Kidney Dis 75(4):S1-64 32778223 10.1053/j.ajkd.2019.12.001

[26] Brunelli SM, Van Wyck DB, Njord L et al (2018) Cluster-randomized trial of devices to prevent catheter-related bloodstream infection. J Am Soc Nephrol 29(4):1336–1343 29472415 10.1681/ASN.2017080870 PMC5875956

[27] Saavedra-Lozano J, Slocker-Barrio M, Fresán-Ruiz E et al (2024) Consensus document of the Spanish Society of Paediatric Infectious Diseases (SEIP) and the Spanish Society of Paediatric Intensive Care (SECIP) for the diagnosis and treatment of central venous catheter-related infections in paediatric care. An Pediatr (Engl Ed) 100(6):448–464 38925786 10.1016/j.anpede.2024.05.012

[28] Mermel LA, Allen M, Bouza E et al (2009) Clinical practice guidelines for the diagnosis and management of intravascular catheter-related infection: 2009 update by the Infectious Diseases Society of America. Clin Infect Dis 49(1):1–45 19489710 10.1086/599376 PMC4039170

[29] Quittnat Pelletier F, Joarder M, Poutanen SM, Lok CE (2016) Evaluating approaches for the diagnosis of hemodialysis catheter-related bloodstream infections. Clin J Am Soc Nephrol 11(5):847–854 27037271 10.2215/CJN.09110815 PMC4858483

[30] Mermel LA, Farr BM, Sherertz RJ et al (2001) Guidelines for the management of intravascular catheter-related infections. Clin Infect Dis 32:1249–1272 11303260 10.1086/320001

[31] Vanholder R, Canaud B, Fluck R et al (2010) Diagnosis, prevention and treatment of haemodialysis catheter-related bloodstream infections (CRBSI): a position statement of European Renal Best Practice (ERBP). Clin Kidney J 3(3):234–246 10.1093/ndtplus/sfq041 PMC6371390 30792802

[32] Warady BA, Same R, Borzych-Duzalka D et al (2024) Clinical practice guideline for the prevention and management of peritoneal dialysis associated infections in children: 2024 update. Perit Dial Int 44(5):303–364 39313225 10.1177/08968608241274096

[33] Heil EL, Bork JT, Abbo LM et al (2021) Optimizing the management of uncomplicated Gram-negative bloodstream infections: consensus guidance using a modified Delphi process. Open Forum Infect Dis 8(10):ofab434 34738022 10.1093/ofid/ofab434 PMC8561258

[34] Farrington CA, Allon M (2019) Management of the hemodialysis patient with catheter-related bloodstream infection. Clin J Am Soc Nephrol 14(4):611–613 30837242 10.2215/CJN.13171118 PMC6450352

[35] Vanholder R, Canaud B, Fluck R et al (2010) Catheter-related bloodstream infections (CRBSI): a European view. Nephrol Dial Transplant 25:1753–1756 20466662 10.1093/ndt/gfq205

[36] Mandolfo S, Possenti S, Lucca B et al (2024) Tunneled hemodialysis central venous catheters prevalence and bloodstream infection rates in Northern Italy: a survey of the “East Lombardy Nephrological Network.” J Vasc Access 25(6):2001–2006 37861341 10.1177/11297298231202081

[37] Zacharioudakis IM, Zervou FN, Arvanitis M et al (2014) Antimicrobial lock solutions as a method to prevent central line-associated bloodstream infections: a meta-analysis of randomized controlled trials. Clin Infect Dis 59(12):1741–1749 25156111 10.1093/cid/ciu671

[38] Sofroniadou S, Revela I, Kouloubinis A et al (2017) Ethanol combined with heparin as a locking solution for the prevention of catheter-related bloodstream infections in hemodialysis patients: a prospective randomized study. Hemodial Int 21(4):498–506 28078825 10.1111/hdi.12524

[39] Nguyen T, Camins BC, Butler DA (2024) Taurolidine and heparin as catheter lock solution for central venous catheters in hemodialysis. Am J Ther 31(4):e398–e409 38710029 10.1097/MJT.0000000000001736

[40] Droste JC, Jeraj HA, MacDonald A, Farrington K (2003) Stability and in vitro efficacy of antibiotic-heparin lock solutions potentially useful for treatment of central venous catheter-related sepsis. J Antimicrob Chemother 51(4):849–855 12654743 10.1093/jac/dkg179

[41] Shanks RM, Sargent JL, Martinez RM et al (2006) Catheter lock solutions influence staphylococcal biofilm formation on abiotic surfaces. Nephrol Dial Transplant 21(8):2247–2255 16627606 10.1093/ndt/gfl170

[42] Lok CE, Appleton D, Bhola C et al (2007) Trisodium citrate 4%—an alternative to heparin capping of haemodialysis catheters. Nephrol Dial Transplant 22(2):477–483 17018541 10.1093/ndt/gfl570

[43] Moran JE, Ash SR, ASDIN Clinical Practice Committee (2008) Locking solutions for hemodialysis catheters; heparin and citrate—a position paper by ASDIN. Semin Dial 21(5):490–492 18764795 10.1111/j.1525-139X.2008.00466.x

[44] Almeida BM, Moreno DH, Vasconcelos V, Cacione DG (2022) Interventions for treating catheter-related bloodstream infections in people receiving maintenance haemodialysis. Cochrane Database Syst Rev 4:CD013554 35363884 10.1002/14651858.CD013554.pub2 PMC8974891

[45] Rosati P, Saulle R, Amato L et al (2021) Mindful organizing as a healthcare strategy to decrease catheter-associated infections in neonatal and pediatric intensive care units: a systematic review and grading recommendations (GRADE) system. J Vasc Access 22(6):955–968 33570016 10.1177/1129729821990215

[46] Bourquelot P, Raynaud F, Pirozzi N (2003) Microsurgery in children for creation of arteriovenous fistulas in renal and non-renal diseases. Ther Apher Dial 7(6):498–503 15018234 10.1046/j.1526-0968.2003.00098.x

[47] Karava V, Jehanno P, Kwon T et al (2018) Autologous arteriovenous fistulas for hemodialysis using microsurgery techniques in children weighing less than 20 kg. Pediatr Nephrol 33(5):855–862 29209823 10.1007/s00467-017-3854-6

[48] Bourquelot P, Van-Laere O, Baaklini G et al (2011) Placement of wrist ulnar-basilic autogenous arteriovenous access for hemodialysis in adults and children using microsurgery. J Vasc Surg 53(5):1298–1302 21276677 10.1016/j.jvs.2010.10.116

