# Supplementary material for: Vascular access for hemodialysis and catheter-related bloodstream infections: a survey on preventive measures and treatment strategies by the EPDWG and ESPN Dialysis Working Group
Source: Eur J Pediatr. 2026 Jan 7;185(1):60. doi: 10.1007/s00431-025-06703-7 (PMC12779675; doi:10.1007/s00431-025-06703-7)
Supplement: Supplementary file 1 — Supplementary file1 (DOCX 18 kb) [file 431_2025_6703_MOESM1_ESM.docx]

**Supplemental Table 1. The survey questions**

| 1 | Your affiliation |
| --- | --- |
|  | Which area are you from? |
|  | How many years have you been practicing in pediatric nephrology? |
|  | What is your current position? |
|  | Which setting do you work in? |
|  | Do you have a pediatric-specific HD unit? |
| 2 | The overall percentage of pediatric chronic HD patients with central venous catheter (CVC)? (from 1 Jan 2021 to31 Dec 2023) |
|  | The overall percentage of chronic HD patients with arteriovenous fistula (AVF)? (from 1 Jan 2021 to 31 Dec2023) |
|  | The overall percentage of chronic HD patients with arteriovenous graft (AVG)? (from 1 Jan 2021 to 31 Dec2023) |
|  | Do you have any HD patient <10 kg (2021 Jan 1st to 2023Dec 31)? |
|  | Do you have any HD patient 10-20 kg (2021 Jan 1st to2023 Dec 31)? |
|  | What is the percentage of central venous catheter (CVC)use in children less than 10 kg? |
|  | What is the percentage of CVC use in children >10-20 kg? (1 Jan 2021- 31 Dec 2023) |
|  | What is the percentage of CVC use in children >20-40 kg? (1 Jan 2021- 31 Dec 2023) |
|  | What is the percentage of central venous catheter (CVC) use in children >40 kg? (1 Jan 2021- 31 Dec 2023) |
|  | Which catheter type do you use in maintenance HD (exclusion from emergency use of uncuffed catheter)? |
|  | Which catheter type do you use in infants? |
|  | What is the main location of permanent CVC for chronic HD? |
| 3 | Which of the followings are the main vascular access-related complications in your center? Please rank them (1: most common) [CVC malfunction, CVC thrombosis, CVC-related bloodstream infection, AVF primary failure, AVF secondary failure] |
|  | Which of the following are the main risk factors for catheter-related bloodstream infections (CRBSI) in your center? |
| 4 | Do you administer IV antibiotics before the insertion of long-term CVCs to prevent CRBSI? |
|  | If yes, which antibiotic? |
|  | How frequent do you perform exit-site cleansing? |
|  | Which topical agents do you use before handling CVC for hand antisepsis? |
|  | Which dressing do you use for CVC exit-site? |
|  | Which topical agents do you use for exit-site antisepsis and dressing change? |
|  | Which antiseptic agents do you use for CVC hubs? |
|  | Do you use needle-free HD connector (Tego, etc) for preventing CVC contamination? |
|  | Do you use chlorhexidine embedded cap (Clear Guard HD antimicrobial barrier cap) device for CVC? |
|  | Do you use exit-site ointments? |
|  | If yes, which topical agent? |
|  | Do you use intranasal mupirocin? |
| 5 | How many cultures do you rely on to diagnose CVC-related bloodstream infection (CRBSI)? |
|  | If you obtain two cultures from CVC and other site, do you use differential time of culture positivity to detect CRBSI? |
| 6 | Which one is your empiric antibiotic selection? |
|  | If methicillin sensitive Staphylococcus aureus was cultured, do you switch to Cefazolin, if glycopeptide was the empiric antibiotic? |
|  | Do you check Vancomycin levels while treating CRBSI? |
|  | Do you check Aminoglycoside levels while treating CRBSI? |
|  | What is the duration of antibiotic treatment for Gr (+) CRBSI? |
|  | What is the duration of antibiotic treatment for Gr (-) CRBSI? |
|  | What are the indications of catheter removal? |
|  | Do you use guidewire exchange procedure in selected cases? |
|  | Do you add antibiotic lock solution (+ anticoagulant) to systemic antibiotics to treat CRBSI as early as possible? |
|  | If yes, which antibiotic lock solution do you use? |
| 7 | Do you routinely use catheter lock solutions? |
|  | If yes, which type locking solutions do you use? |
|  | If single agent is used, which one is used in routine practice? |
|  | If double/triple agents are used, which one is used in routine practice? |
|  | If 2+1 protocols are used, which one is used in routine practice? |
|  | Do you use anti-fungal prophylaxis during treatment of bacterial CRBSI? |
|  | If yes, which agent? |
| 8 | How many infections did you record last 3 years ( 1 Jan 2021- 31 Dec 2023) |
|  | Total HD patient number: |
|  | Number of the patients dialyzed with permanent cuffed CVC: |
|  | No of exit-site infection: |
|  | No of CRBSI: |
|  | Number of CVC exchange: |
|  | Number of CVC Exchange due to CRBSI: |
|  | Number of CVC exchange due to exit-site infection: |
